# Editorial: Brain imaging relations through simultaneous recordings

**DOI:** 10.3389/fnins.2023.1139336

**Published:** 2023-02-07

**Authors:** Waldemar Karwowski, Surjo R. Soekadar, Aleksandra Kawala-Sterniuk

**Affiliations:** ^1^Department of Industrial and Systems Engineering, University of Central Florida, Orlando, FL, United States; ^2^Clinical Neurotechnology Laboratory, Department of Psychiatry and Neurosciences, Charité Campus Mitte (CCM), Charité–Universitätsmedizin Berlin, Berlin, Germany; ^3^Faculty of Electrical Engineering, Automatic Control and Informatics, Opole University of Technology, Opole, Poland

**Keywords:** neural technology, MRI, EEG, fMRI, functional near-infrared spectroscopy, magnetoencephalography, simultaneous recordings

## 1. Introduction

Recent technological advancements in neuroscience and in particular neuroimaging has improved the understanding of brain functionality and connectivity—not only in animal but also in human brains (Kawala-Sterniuk et al., 2021; Saeidi et al., 2021; Simon et al., 2021). Today, we are not only able to identify, localize, and characterize brain pathologies, such as local infections, lesions, or tumors, but we are also able to study brain anatomy, functionality, development, neuronal networks, etc. with very high precision. Availability of sophisticated software and advanced algorithms improved the possibilities for the analysis of various biomedical signals and outputs (Ismail and Karwowski, 2020; Kawala-Sterniuk et al., 2021; Martinek et al., 2021).

Despite the above-mentioned rapid technological development in this field, certain machine-specific limitations have to be taken into consideration, as these affect our ability to fully understand the mechanisms underlying brain functions. The limitations include poor temporal and poor spatial resolution, as each respective neuroimaging technique is accompanied by some shortcomings. Hence, the recent shift in the field has led to hybrid methods, which are a combination of simultaneous neuroimaging methods such as electroencephalography (EEG) and magnet resonance imaging (MRI) or functional near-infrared spectroscopy (fNIRS). Such combined approaches overcome the limitations of single modalities and provide a fuller and more comprehensive picture of the brain. They also play an increasing role when interacting with the brain using stimulation techniques (e.g., transcranial electric or magnetic stimulation (TES/TMS), including closed-loop applications) (Nasr et al., 2022).

The Research Topic “*Brain Imaging Relations Through Simultaneous Recordings*” consists of a collection of 11 contributions discussing new methods and systems for various brain data recordings and analyses, and document the most recent advancements in the field of neural technology and simultaneous neuroimaging recordings.

The first article published by Żebrowska et al. titled “*Removal of the Sinusoidal Transorbital Alternating Current Stimulation Artifact From Simultaneous EEG Recordings: Effects of Simple Moving Average Parameters*” focuses on the analysis of electroencephalography (EEG) signals, which is a very challenging task—mostly due to the nature of these signals. This study states that alternating current stimulation can be a promising treatment method for various neurological disorders, but it causes numerous artifacts and disturbances in EEG signals. In order to remove these artifacts, the authors proposed a simple moving average subtraction, which gave very promising and positive results. The authors of that paper based on a thorough literature background, proved that moving average filtering can be efficiently applied in EEG signals' processing.

The second article, entitled “*Reliability of MUSE 2 and Tobii Pro Nano at capturing mobile application users' real-time cognitive workload changes*,” was written by Zhang and Cui. Unlike Żebrowska et al., this study focused on non-clinical, inexpensive equipment such as MUSE 2 in order to check if it could be a useful tool for obtaining high-quality EEG signals–potentially later applied for diagnostics purposes (Zhang and Cui). The article is also focused on a very broad topic—human omputer interaction (HCI), where the MUSE 2-EEG was combined with an eye-tracking device (Tobii Pro Nano). The obtained signals were of high quality and stable, making them useful in studies assessing the usability of smartphone applications. Despite some flaws, the authors have proven MUSE 2 to be a reliable device for cognitive workload detection and measurement.

Functional near-infrared spectroscopy (fNIRS) can be considered a less expensive alternative to fMRI functional magnetic resonance imaging). The use of fNRIS has been discussed in two articles: Deng et al. and Ma et al.. In the first article written by Deng et al., titled “*The analgesic effect of different interactive modes of virtual reality: A prospective functional near-infrared spectroscopy (fNIRS) study*,” the authors focused on studying the analgesic effect of various virtual reality (VR) models with the use of fNIRS measurements. The obtained results proved VR to have the analgesic effect, which has been verified by the analysis of fNIRS signals. The second article written by Ma et al. and titled “*Increased cerebral cortex activation in patients with stroke during the electrical stimulation of cerebellar fastigial nucleus with functional near-infrared spectroscopy*” applied fNIRS data in order to detect any functional connectivity changes in patients affected by brain stroke and to study the cortical activation caused by fastigial nucleus (FNS) by measuring the cerebral cortex oxygenated hemoglobin concentration (HBO), which can be done with the fNIRS of both patients with stroke and healthy controls. This study proved that combining FNS and fNIRS techniques can help in choosing appropriate functional rehabilitation for patients with stroke.

Electroencephalography recordings can be efficiently combined with fNIRS as presented in article written by Chen et al. titled “*Amplitude of fNIRS resting-state global signal is related to EEG vigilance measures: A simultaneous fNIRS and EEG study*” describing fNIRS-EEG simultaneous recordings in healthy participants. The presented study consisted of two experiments, where the first one was carried out on patients in the supine, sitting, and standing positions; while the second experiment concerned the analysis of fluctuations between the epochs of a separate group of subjects. The authors found a negative temporal correlation between EEG vigilance measurements and global fNIRS signal amplitudes. According to the authors, this is the first study to reveal that vigilance as a neurophysiological factor modulates fNIRS dynamics at rest, which has important implications for understanding and processing noise in fNIRS signals.

Three studies using hybrid recording systems based on EEG and fMRI (functional magnetic resonance imaging) are also part of this special issue: Bhutada et al., Rusiniak et al., and Basedau et al.. The first article written by Bhutada et al., titled “*Semi-automated and direct localization and labeling of EEG electrodes using MR structural images for simultaneous fMRI-EEG*” describes both semi-automated and direct methods for standard EEG cap electrode localization and labeling. The data were obtained during simultaneous fMRI-EEG recordings. The authors proposed a novel, semi-automated method as a simple alternative for rapid electrodes labeling and localization with no need for using any additional equipment than the one already applied in a typical EEG-fMRI setup. Rusiniak et. al. wrote an article (“EEG-fMRI: Ballistocardiogram Artifact Reduction by Surrogate Method for Improved Source Localization”), where they focused on ballistocardiogram (BCG) removal from the brain signals obtained during EEG-fMRI recording. It is because the other biosignals are frequently acting and considered artifacts while present in brain recordings. Removal of such distorting biomedical signals is a very challenging task. Rusiniak et al. proposed a method based on surrogate source models applied for the purpose of artifact removal with the possible minimal distortion. In the third article written by Basedau et al.—“*High-density electroencephalography-informed multiband functional magnetic resonance imaging reveals rhythm-specific activations within the trigeminal nociceptive network*,” the authors focused on using multi-modal non-invasive imaging techniques for the pain assessment purposes. The authors showed that changes in theta-band visible in EEG recordings are induced by trigeminal pain and these correlate with fMRI activation in the brainstem.

In another article, brain data obtained using imaging techniques were applied to assess the severity of pain Wang et al.. As cancer affects an increasing number of people, one of its most common symptoms is cancer pain (CP), which frequently reduces life quality. In an article written by Wang et al. titled “*Evaluation of the glymphatic system with diffusion tensor imaging-along the perivascular space in cancer pain*,” the authors decided to apply diffusion tensor imaging along the perivascular space (DTI-ALPS) as a non-invasive method to detect the alteration of the caused by bone metastasis glymphatic function in patients affected with CP. Their findings can improve understanding not only the functional characteristics of the brain under cancer pain but also to evaluate it through brain function detection, which may play a crucial role in appropriate treatment formulation. Neuroimaging techniques described in that article may be considered biomarkers for cancer pain assessment.

Our special issue also contains a research article titled “Evaluating the Safety of Simultaneous Intracranial Electroencephalography and Functional Magnetic Resonance Imaging Acquisition Using a 3 Tesla Magnetic Resonance Imaging Scanner” by Fujita et al.. It presents a fMRI-based multi-modal system that was combined with invasive brain activity recordings–intracranial electroencephalography (icEEG). Due to the invasiveness of icEEG recording, both methods have never been carried out at the same time. The authors of this study decided to conduct both measurements simultaneously using a 3-Tesla scanner. The authors considered major risk factors and assessed safety rules. Their study proved that under appropriate conditions that health risks during such procedure are low.

The review article titled “*Vascular cognitive impairment after mild stroke: connectomic insights, neuroimaging, and knowledge translation*” written by Holguin et al. underlines that current stroke assessment protocols rarely detect vascular cognitive impairment (VCI), in particular among patients affected with lighter deficits. The authors emphasize the importance of screening for VCI because such screening provides information required for the rehabilitation and recovery process. In this article, the authors focused on the relationship between insult-induced connectome changes and the VCI; and discussed the latest clinical approaches to identify disruptions in neural networks and white matter connectivity. It also outlines how occupational therapists can work to make significant clinical innovations and speed recovery for people affected by stroke.

As mentioned earlier, analysis of biomedical data, in particular brain signals, is a very challenging task, but this makes it very interesting. We hope that our Research Topic will be found interesting to readers and researchers in fields of medicine, biomedical engineering, or neuroscience.

## Author contributions

All authors listed have made a substantial, direct, and intellectual contribution to the work and approved it for publication.

## References

[B1] Ismail L. E. Karwowski W. (2020). Applications of EEG indices for the quantification of human cognitive performance: a systematic review and bibliometric analysis. PLoS ONE 15, e0242857. 10.1371/journal.pone.0242857 33275632 PMC7717519

[B2] Kawala-Sterniuk A. Browarska N. Al-Bakri A. Pelc M. Zygarlicki J. Sidikova M.. (2021). Summary of over fifty years with brain-computer interfaces review. Brain Sci. 11, 43. 10.3390/brainsci11010043 33401571 PMC7824107

[B3] Martinek R. Ladrova M. Sidikova M. Jaros R. Behbehani K. Kahankova R.. (2021). Advanced bioelectrical signal processing methods: Past, present and future approach part II: brain signals. Sensors 21, 6343. 10.3390/s21196343 34640663 PMC8512967

[B4] Nasr K. Haslacher D. Dayan E. Censor N. Cohen L. G. Soekadar S. R. (2022). Breaking the boundaries of interacting with the human brain using adaptive closed-loop stimulation. Prog. Neurobiol. 216, 102311. 10.1016/j.pneurobio.2022.102311 35750290

[B5] Saeidi M. Karwowski W. Farahani F. V. Fiok K. Taiar R. Hancock P.. (2021). Neural decoding of eeg signals with machine learning: a systematic review. Brain Sci. 11, 1525. 10.3390/brainsci11111525 34827524 PMC8615531

[B6] Simon C. Bolton D. A. Kennedy N. C. Soekadar S. R. Ruddy K. L. (2021). Challenges and opportunities for the future of brain-computer interface in neurorehabilitation. Front. Neurosci. 15, 699428. 10.3389/fnins.2021.699428 34276299 PMC8282929

